# Unintended Epidural Spread Following Unilateral Erector Spinae Plane (ESP) Block: A Case Report

**DOI:** 10.7759/cureus.87888

**Published:** 2025-07-14

**Authors:** Diego Fiume, Michele Arciuolo, Beatrice Baldelli, Silvia Carlini, Massimo Galletti

**Affiliations:** 1 Department of Anesthesiology and Critical Care, UniCamillus - Saint Camillus International University of Health and Medical Sciences, Rome, ITA; 2 Department of Anesthesiology, Sant’Eugenio Hospital, Rome, ITA; 3 Department of Anesthesiology, Tor Vergata University, Rome, ITA; 4 Department of Anesthesiology, Azienda Ospedaliera S. Giovanni Addolorata, Rome, ITA

**Keywords:** breast surgery, erector spinae plane, esp, espb, locoregional anesthesia

## Abstract

The erector spinae plane block (ESPB) is a fascial block that consists of the injection of local anesthetic into the space between the erector spinae muscle and the transverse process of the vertebrae. The clinical case presented concerns the execution of an ESPB to control perioperative pain in a patient undergoing breast surgery and the side effects we encountered. Although the block is very effective and easy to perform, it has unpredictable side effects, which are still the subject of numerous clinical studies. ESPB is used all over the world; it is important to know more about the complications that can occur with this block. This case report aims to improve knowledge in this regard.

## Introduction

The erector spinae plane block (ESPB) is a fascial block described for the first time in 2016 by Forero et al., who used it to treat pain in two patients with rib fractures [1]. It is currently widely used in perioperative analgesia in thoracic and abdominal surgery and the treatment of chronic painful pathologies [2-5]. The technique, generally ultrasound-guided, is easy to perform and consists of the injection of local anesthetic into the space between the erector spinae muscle and the transverse process of the vertebrae. *Erector spinae* is a group of posterior paraspinal muscles that runs from the base of the skull to the sacral region, and, for this reason, ESPB can be performed at any level of the spine. Despite its versatility and few contraindications (patient refusal and infection at the injection site), it is still the subject of numerous studies investigating its mechanism of action [2]. Although not yet fully known, these appear to consist primarily of blockade of the ventral and dorsal branches of the spinal nerves; recent studies, carried out on cadavers and with the aid of finer imaging techniques such as magnetic resonance imaging, have demonstrated a spread of the anesthetic in the cranio-caudal direction of at least three to four spaces from the injection site [3]. Furthermore, in a completely unpredictable way, there would appear to be a reabsorption of the drug in the epidural space, which would explain some complications reported in the literature, such as sudden hypotension and muscle block [6-9].

## Case presentation

A 50-year-old Caucasian woman (56 kg, 165 cm) was scheduled for breast expander removal and reconstruction with a prosthesis. About six months earlier, the patient had undergone a left radical mastectomy, following a diagnosis of breast cancer. At the time of the operation, she was in fair general condition, and the medical history was negative for other noteworthy pathologies and allergies.

**Figure 1:**
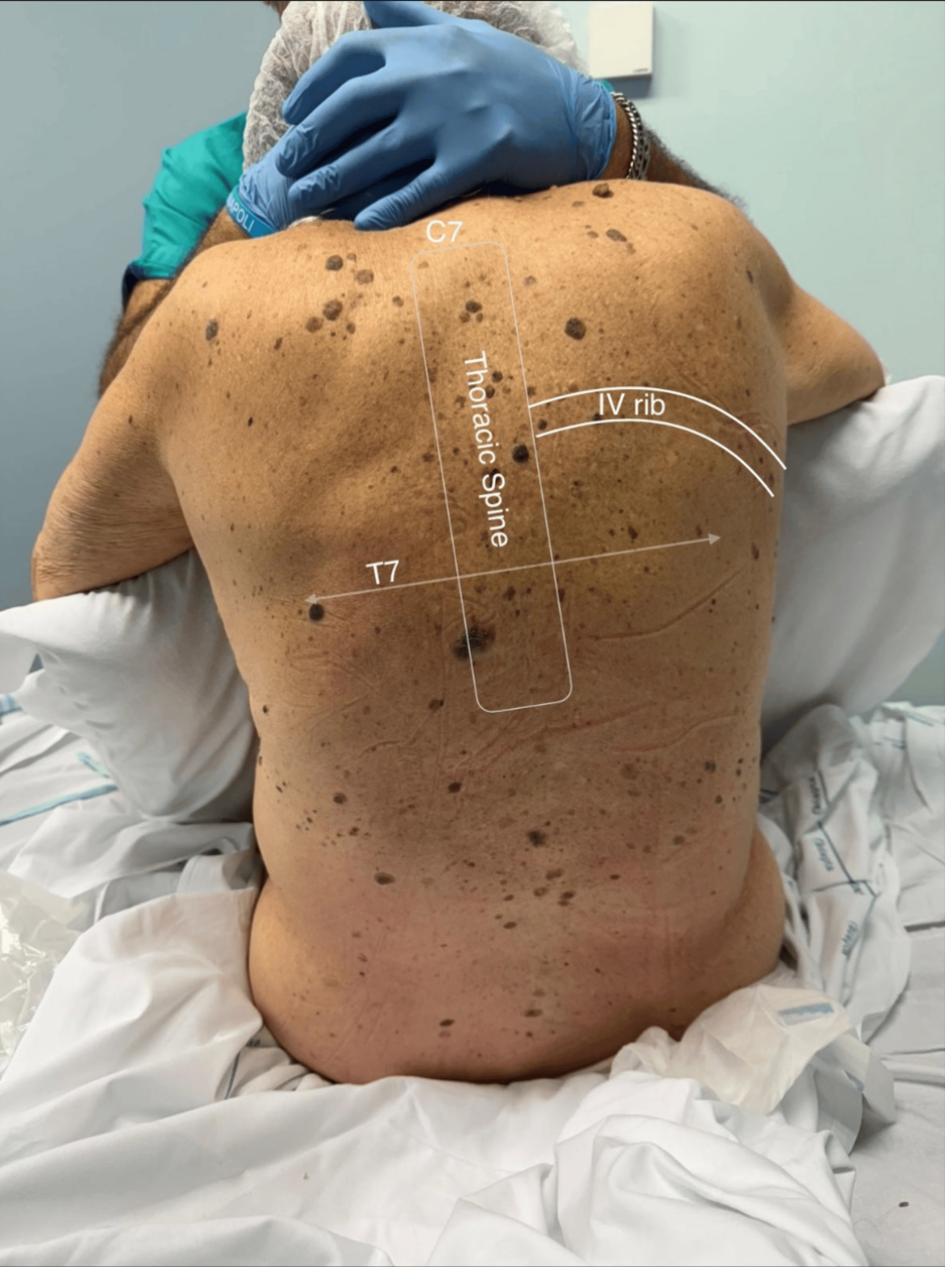
Anatomy in the sitting position: C7 (seventh cervical vertebra), T7 (seventh thoracic vertebra), and IV rib (fourth rib).

**Figure 2:**
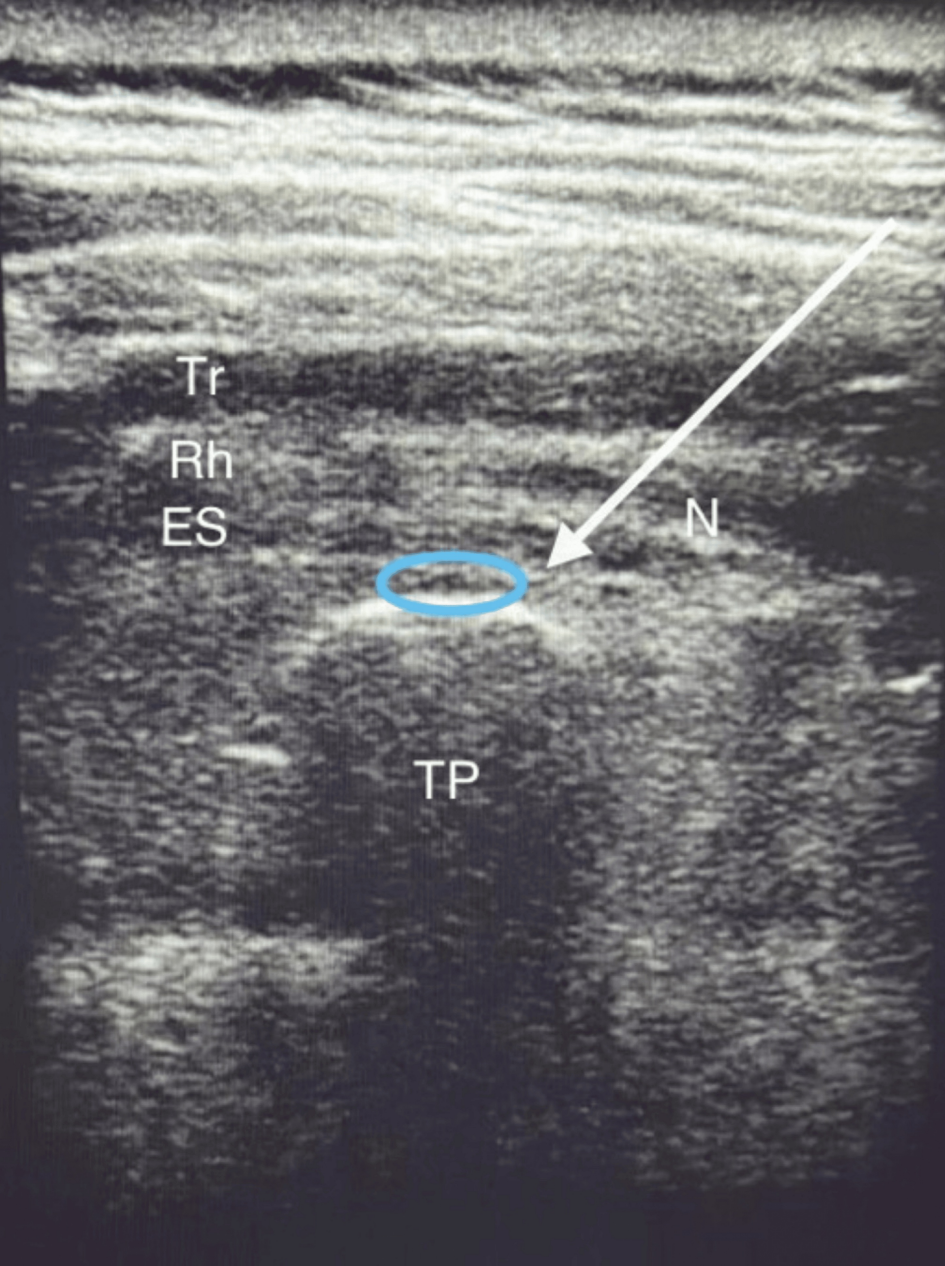
US anatomy for ESP block. US, ultrasound; ESP, erector spinae plane; Tr, trapezius; Rh, rhomboid; ES, erector spinae; TP, transverse process; N, needle; colored area, landing point

In consideration of the favorable clinical conditions and with the patient's consent, it was decided to perform unilateral ultrasound-guided ESPB for perioperative pain control. Monitored and in a sitting position (Figure 1), we proceeded with ultrasound visualization of the fascial plane at the T4 level and with in-plane (Figure 2), cranio-caudal injection of Levobupivacaine 0.25% and Mepivacaine 1%, for optimal duration of action and decreased onset. A total of 30 mL of mixture was injected, in line with the dose range described in the literature for unilateral ESPB [3]. We used a 22 G 80 mm needle and a high-frequency linear probe. 

After approximately 20 minutes, the patient manifested a sudden and worsening sense of agitation, associated with respiratory difficulty, tachypnea (respiratory rate 30 apm), and tachycardia (heart rate 125 bpm), for which Midazolam 5 mg IV was initially administered, but did not result in a resolution of the condition. For this reason, induction of general anesthesia and oro-tracheal intubation were performed. Throughout the entire duration of the operation, which lasted approximately 3 hours, the patient remained hemodynamically stable, mean arterial pressure never fell below 65 mmHg, and was maintained with total intravenous anesthesia (TIVA) with Propofol 2%, approximately 6-7 mL/kg/hour.

Upon awakening, the patient presented with muscular weakness in the upper limbs bilaterally (left>right), with difficulty in raising and flexing them, and reported that she remembered difficulty in expanding the chest before induction. Given the clinical case compatible with an epidural spread of local anesthetic, she was then transferred to the recovery room for postoperative monitoring, during which she remained clinically stable. We observed no pain (Numerical Rating Scale (NRS) = 0) and complete resolution of the limb strength deficit five hours after the block was performed. After another two hours of clinical monitoring, the patient was discharged from the recovery room.

## Discussion

The erector spinae muscle is composed of three muscle groups: a lateral group (iliocostalis), an intermediate group (longissimus), and a medial group (spinalis). It is a deep muscle that extends from the cervical spine to the sacrum and is essential for maintaining an upright posture [2,3]. Deep within the erector spinae, we find the group of transversospinal muscles (semispinalis, multifidus, and rotators) that connect the transverse and spinous processes, and even deeper, small muscles that connect the transverse processes to the ribs. The spinal nerves run through this complex muscular network, consisting of the union of a ventral and a dorsal branch, which emerge from the spinal cord and join at the level of the intervertebral foramen; the dorsal branch ascends through the thoracolumbar fascia and erector spinae muscle to the superficial tissues, while the ventral branch continues along the inner caudal surface of the rib as the intercostal nerve, giving off collateral branches to muscles and bones along the way [3].

As already mentioned, the ESPB consists of the ultrasound-guided injection of local anesthetic underneath the muscle itself and superficially to the transverse process. In practice, with the patient in a sitting position, a high-frequency linear probe is positioned approximately 3 cm lateral to the spine to visualize the transverse process, which appears hyperechoic, along with the erector spinae, rhomboid major, and trapezius muscles. Once the target vertebral level is identified, the drug is injected in a cranio-caudal direction [3,4].

In recent years, the use of ESPB has expanded and is now employed not only in the treatment of chronic pain conditions, such as spinal disorders, but also in perioperative pain management for major surgeries, particularly thoracic and abdominal procedures [2-5]. The ease of execution and its safety, given the small number of complications documented in the literature, make this block very versatile and effective from an analgesic point of view [5].

The use of interfascial plane blocks, particularly in breast surgery, has demonstrated several advantages: reduced postoperative pain, decreased opioid requirements, a lower risk of nausea, vomiting, and pulmonary complications, and reduced ICU stays. Furthermore, ESPB has shown analgesic effectiveness comparable to other interfascial plane blocks, such as the paravertebral and serratus blocks [5,10]. However, it still remains to be clarified the exact mechanism of action of the anesthetic, the unclear diffusion of which can cause unpredictable collateral, as in the clinical case in question.

Several studies have, therefore, tried to understand the exact mechanism of action, in particular by observing the diffusion of the drug in human cadavers, via MRI. As reported in these studies [11-14], the diffusion of the anesthetic seems to proceed in three main directions: cranio-caudally, laterally, and anteriorly. The cranio-caudal spread probably depends on the quantity of drug injected and extends for at least three to four levels compared to the starting one; a portion penetrates the paravertebral space anteriorly, bathing the dorsal rami of the spinal nerves, while laterally it remains confined below the thoracolumbar fascia. Furthermore, a minimal fraction of anesthetic diffuses into the epidural space. Penetration into the epidural space seems to contribute to the effect of the block and strengthen its analgesic efficacy. Some authors have proposed it as an alternative to epidural anesthesia for the control of postoperative pain in thoracic surgery [9]. Although only a few milliliters of anesthetic reach the epidural space, it is practically impossible to confirm this by ultrasound. For this reason, completely unpredictable side effects may occur, as reported in some cases in the literature. Some anesthetists have encountered episodes of hypotension and bilateral muscle weakness, particularly in the lower limbs, after performing ESPB in both the thoracic and lumbar regions [6-8,15].

In our case, although the ultrasound visualization and the needle position were optimal, the patient showed a sudden and worsening sense of agitation approximately 20 minutes after performing the block, associated with respiratory difficulty and tachycardia. In the immediate postoperative period, there was also bilateral muscular weakness in the upper limbs, associated with difficulty in raising and flexing them, 3/5 motor strength (movement possible, against gravity too). The dermatomal disposition and respiratory difficulty without additional neurological symptoms, such as seizures, did not lead us to suspect other clinical conditions (e.g., local anesthetic systemic toxicity). Likewise, the bilateral nature of the symptoms, the lack of stiffness and pain in the neck and head, led us to exclude cervical pathology and other pathologies with a mainly unilateral manifestation. The state of anxiety seemed unlikely to us: the onset time of 20 minutes and the lack of response to the administration of Midazolam 5 mg IV made us think of other diagnostic hypotheses. Lastly, a possible allergic reaction was ruled out either as the patient had no altered tests, the anamnesis was silent, and she had no other suggestive clinical cutaneous, respiratory, or hemodynamic manifestations.

In consideration of these clinical data, the volume of the anesthetic, and the high level (T4) at which it was injected, we can hypothesize that there was not only a spread in the cranial direction, but also an infiltration into the epidural space, confirmed by the bilaterality of the postoperative motor block. The dosage of the anesthetic mixture was not low enough to provide adequate analgesic coverage of the entire surgical incision. Furthermore, at the T4 level, the epidural space narrows considerably. This clinical case suggests an inadvertent spread of local anesthetic into the epidural space, and the symptoms could be compatible with this hypothesis.

The resolution of these side effects occurred within a few hours; the patient remained hemodynamically stable both during the operation and postoperatively, and, most importantly, experienced no pain.

## Conclusions

The ESPB is now considered one of the most effective interfascial plane blocks for controlling perioperative pain; especially in recent years, various studies have been conducted, many still ongoing, to better understand its mechanism of action. However, the ease of execution and effectiveness should not make operators underestimate the unpredictability of its effects. As we found with our case, the correctness of the procedure does not exclude possible complications. In conclusion, our clinical case would like to contribute to remembering how an analgesic block, considered easy to perform, can seriously compromise the health and safety of the patient.
